# Decreasing Phosphatidylcholine on the Surface of the Lipid Droplet Correlates with Altered Protein Binding and Steatosis

**DOI:** 10.3390/cells7120230

**Published:** 2018-11-24

**Authors:** Laura Listenberger, Elizabeth Townsend, Cassandra Rickertsen, Anastasia Hains, Elizabeth Brown, Emily G. Inwards, Angela K. Stoeckman, Mitchell P. Matis, Rebecca S. Sampathkumar, Natalia A. Osna, Kusum K. Kharbanda

**Affiliations:** 1 Departments of Biology and Chemistry, St. Olaf College, Northfield, MN 55057, USA; liz.townsend@wisc.edu (E.T.); cassie.rickertsen@gmail.com (C.R.); anahains@gmail.com (A.H.); elizabeth.hadley94@gmail.com (E.B.); 2 Department of Chemistry, Bethel University, St. Paul, MN 55112, USA; egi25796@bethel.edu (E.G.I.); a-stoeckman@bethel.edu (A.K.S.); 3 Research Service, VA Nebraska-Western Iowa Health Care System, Omaha, NE and Departments of Internal Medicine and Biochemistry & Molecular Biology, University of Nebraska Medical Center, Omaha, NE 68105, USA; mitchell.matis@unmc.edu (M.P.M.); rebecca.sampathkumar@unmc.edu (R.S.S.); nosna@unmc.edu (N.A.O.); kkharbanda@unmc.edu (K.K.K.)

**Keywords:** lipid droplets, perilipins, phosphatidylcholine, phosphatidylethanolamine, steatohepatitis

## Abstract

Alcoholic fatty liver disease (AFLD) is characterized by an abnormal accumulation of lipid droplets (LDs) in the liver. Here, we explore the composition of hepatic LDs in a rat model of AFLD. Five to seven weeks of alcohol consumption led to significant increases in hepatic triglyceride mass, along with increases in LD number and size. Additionally, hepatic LDs from rats with early alcoholic liver injury show a decreased ratio of surface phosphatidylcholine (PC) to phosphatidylethanolamine (PE). This occurred in parallel with an increase in the LD association of perilipin 2, a prominent LD protein. To determine if changes to the LD phospholipid composition contributed to differences in protein association with LDs, we constructed liposomes that modeled the LD PC:PE ratios in AFLD and control rats. Reducing the ratio of PC to PE increased the binding of perilipin 2 to liposomes in an in vitro experiment. Moreover, we decreased the ratio of LD PC:PE in NIH 3T3 and AML12 cells by culturing these cells in choline-deficient media. We again detected increased association of specific LD proteins, including perilipin 2. Taken together, our experiments suggest an important link between LD phospholipids, protein composition, and lipid accumulation.

## 1. Introduction

Fatty liver is an early and consistent morphological feature of alcohol-related liver injury, and with continued alcohol consumption, can progress to cirrhosis and liver failure [1,2]. The magnitude of this problem is notable; alcohol-related liver cirrhosis was responsible for nearly 500,000 deaths worldwide in 2010 [3]. Despite widespread recognition of this significant health problem, the mechanism of how excessive alcohol consumption results in liver steatosis remains unclear.

With the aim of identifying factors that contribute to fatty liver, we characterized hepatic lipid droplets (LDs) in a rat model of alcoholic liver injury. LDs are composed of a core of neutral lipids including triglycerides and cholesteryl esters. The neutral lipid core of an LD is shielded from the surrounding cytosol by a phospholipid monolayer consisting mostly of phosphatidylcholine (PC) and phosphatidylethanolamine (PE) [4,5]. The LD surface is further decorated with LD-associated proteins, the functions of which vary widely and include roles in lipid metabolism, trafficking, and signaling [6,7,8]. Identification of the mechanisms that target LD proteins to the LD surface is an emerging area of research [9].

Given the intimate relationship between LD proteins and LD function, efforts have been made to document changes to the LD proteome with the onset of alcohol-induced hepatic steatosis [10,11]. Several studies have reported altered levels of perilipin 2, an abundant and ubiquitously expressed member of the perilipin protein family. Five major perilipin proteins are known in mammals. While there are differences in tissue-specific patterns of expression and regulation, perilipin proteins are widely known to promote lipid storage in LDs [12]. Perilipin 2 correlates with lipid accumulation in multiple tissues [13] and likely plays an important role in facilitating overaccumulation of lipids in the liver. Elevated levels of perilipin 2 have been detected in both human liver biopsies [14] and animal models of alcoholic fatty liver disease (AFLD) [15,16,17,18]. In addition, the absence of perilipin 2 in mice prevents alcohol-induced [19] or diet-induced [20] hepatic steatosis. 

Significant changes to the lipid composition of hepatocytes have been observed during the development of steatosis. Fernando et al. reported differences in the levels of hepatic cholesterol, triglycerides, and phospholipids that distinguish alcohol-fed rats from paired control animals [21]. We and others have detected a decrease in hepatic PC levels following alcohol ingestion [22,23,24]. Studies from our laboratory have implicated alcohol in the alteration of the methionine metabolic pathway [25,26] and the consequent lowering of the ratio of hepatic S-adenosylmethionine (SAM) to S-adenosylhomocysteine (SAH) [23]. The decrease in this ratio is known to inhibit the activities of many methyltransferases [25,26] including phosphatidylethanolamine methyltransferase (PEMT) [23,27]. Because PEMT catalyzes the three successive methyl group transfers from SAM to PE to generate PC [28], inhibition of this enzyme decreases PC synthesis. 

In this study, we found significant differences in the overall composition of hepatic LDs from control and ethanol-fed rats. Relative amounts of PC and PE, along with association of specific LD proteins, were altered in hepatic LDs from ethanol-fed rats. We modeled the changes to the phospholipid composition of LDs in cultured cells and in vitro experiments and detected similar changes in the association of specific LD proteins. Therefore, we propose that changes to the LD phospholipid composition impact protein association and LD function. 

## 2. Materials and Methods

### 2.1. Diet Formulation

Nutritionally adequate Lieber–DeCarli control and ethanol liquid diets [29] were purchased from Dyets, Inc. (Bethlehem, Palestine). The ethanol diet consisted of 18% total energy as protein, 35% as fat, 11% as carbohydrate, and 36% as ethanol. In the control diet, ethanol was replaced isocalorically with carbohydrate such that both ethanol and control rats consumed identical amounts of all nutrients except carbohydrate.

### 2.2. Ethanol Feeding Procedure 

Male Wistar rats (Charles River Laboratories, Wilmington, MA, USA) weighing 180–200 g (approximately 45–48-days old) were weight-matched and divided into two groups. Group 1 was fed the control diet and group 2 was fed the ethanol diet. The rats were pair-fed in that the group 1 rats were fed the amount of diet consumed by the rats in group 2 on the previous day. Overall, each group consisted of eight rats fed the appropriate diet for 5–7 weeks. Twenty-four hours before sacrifice, the total daily volume of the diet was divided with one-fourth given at 8:00 am, one-fourth at 12:00 pm, and one-half at 4:00 pm. In addition, animals were given one-fourth of their respective diets 60–90 min prior to sacrifice. This regimen was followed to minimize differences in feeding patterns that exist between the groups of rats. The care, use, and procedures performed were approved by the Institutional Animal Care and Use Committee of the Omaha Veterans Affairs Medical Center and were in accordance with the Public Health Service (PHS) Policy on Human Care and Use of Laboratory Animals. 

### 2.3. Histological Analysis of LD Number and Size

Tissues were frozen in optimum cutting temperature compound (Tissue-TeK, Torrance, CA, USA). Fresh cut sections (5 μm) were fixed in 4% *w/v* paraformaldehyde in 50 mM PIPES, pH 7.0, and the accumulated lipid was visualized by staining with 1 µg/mL BODIPY 493/503 (Invitrogen, Carlsbad, CA, USA). After incubation, slides were washed twice with PBS and mounted with Vectashield/DAPI (Vector laboratories, Burlingame, CA, USA). Images were obtained with a Zeiss 510 Meta Confocal Laser Scanning Microscope using an excitation wavelength of 488 nm and an emission wavelength of 505 nm. Quantification of LD number and size was carried out using ImageJ software (NIH, Bethesda, MD, USA). For quantification, three different fields were randomly selected from each section and data were pooled from sections obtained from three different sets of control and ethanol-fed animal pairs. 

### 2.4. Lipolysis

Isolated hepatocytes were plated on collagen-IV-coated dishes using Williams’ E medium supplemented with antibiotics and 5% FCS. After 2 h of plating, the growth medium was replaced with Williams’s E without serum medium supplemented with 50 µM oleic acid complexed to 0.4% BSA containing [^3^H]-oleic acid (1 × 10^6^ dpm/dish) as a tracer to label cellular triglycerides. To examine cellular lipolysis, the efflux of free [^3^H]-oleic acid was tracked over 6 h in the presence of 10 µM triacsin C (Biomol, Plymouth Meeting, PA, USA), an inhibitor of acylcoenzyme A synthetase in the medium that prevents re-esterification of fatty acids.

### 2.5. Isolation of LDs from Rat Livers

LDs were isolated from rat liver as described previously [18]. Briefly, 20% liver homogenate was prepared in 60% sucrose in TE buffer (10 mM Tris-HCl, 1 mM EDTA, pH 7.4) containing 1 mM PMSF and protease inhibitor cocktail. A postnuclear supernatant (PNS) fraction was obtained by centrifugation (1000 × *g*) of the homogenate for 10 min. LDs were isolated by subjecting the obtained PNS to ultracentrifugation through prechilled discontinuous gradient of sucrose solutions (40%, 25%, and 10%) in TE buffer. The tubes were centrifuged (30,000 × *g*) in a precooled SW28 rotor in a Beckman L-70 ultracentrifuge at 4 °C for 30 min with slow acceleration and no brake. The white band (LD fraction) at the top of the gradient was collected and further purified by centrifugation (20,800 × *g*) for 10 min. The clear buffer underlying the white band was removed and the LD fraction diluted to 200 μL with TE buffer and frozen at −70 °C. The total volume of reconstitution buffer remained constant/gm liver used for homogenization. 

### 2.6. SDS-PAGE and Immunoblotting of Proteins from Rat Livers

Western blotting was performed as described previously [23,30]. Briefly, LD samples (diluted 1:4) were resolved on 12% SDS-PAGE under reducing conditions and transferred onto nitrocellulose membranes. The blots were blocked for 1 h at 37 °C with PBS, pH 7.4, 0.05% Tween-20 (PBS-T) containing 5% nonfat dry milk and then incubated overnight with primary antibodies at 4 °C. Anti-Perilipin 2 (cat# 10R-A117ax; Fitzgerald, Acton, MA, USA) and Anti-Perilipin 3 (cat# ab77365; Abcam Inc., Cambridge, MA, USA) were used at a 1:20,000 and 1:2,000 dilution, respectively. After incubation with the primary antibodies, the membranes were washed 3 times for 5 min each with PBS-T and then incubated with peroxidase-conjugated secondary antibody for 1 h at room temperature. Blots were extensively washed with PBS-T, washed once with PBS, and the proteins were visualized using standard ECL detection methods. The intensities of immunoreactive protein bands were quantified using Quantity One software (Bio-Rad Laboratories, Hercules, CA, USA). 

### 2.7. Lipid Analysis from Rat Livers

Lipids were extracted from liver tissue, or isolated hepatic LDs, using the Folch procedure [31]. The total triglyceride content from these samples was determined as detailed in [23]. In addition, the lipid residue was dissolved in chloroform and applied (along with appropriate standards) to a Silica gel G-plate (Uniplate, Analtech Inc., Newark, DE, USA) to separate PC and PE by TLC using n-propyl alcohol/propionic acid/chloroform/water (2:2:2:1) as solvent [32]. The spots corresponding to PC and PE, verified by standards run on the same plate, were visualized by spraying with Molybdenum Blue (Sigma-Aldrich, St. Louis, MO, USA), followed by scraping and subsequent analysis for total phosphorous [33] to quantify the level of PC and PE.

### 2.8. Hepatic S-adenosylmethionine-to-S-adenosylhomocysteine (SAM:SAH) Ratio 

High-performance liquid chromatography (HPLC) analysis was performed on the perchloric acid extract of total liver for determining SAM and SAH levels as detailed previously [21] to calculate the SAM:SAH ratio.

### 2.9. In Vitro Binding Assay

Perilipin 2 association with large unilamellar vesicles (LUVs) of varied phospholipid composition was performed as previously described [34]. Briefly, a total of 12 μmols of dioleoyl-PC and dioleoyl-PE (Avanti Polar Lipids) at ratios of 4.5:1, 3.5:1, 2.5:1, and 1.5:1 were dried under vacuum. Samples were warmed to 70 °C and 1 mL of Hepes/EDTA buffer (10 mM Hepes, 5 mM EDTA, pH 7.4) was added to each. Lipid films were hydrated on a rocking platform at 50°C. LUVs were formed by five cycles of freeze/thaw in a dry ice and ethanol bath. 

The cytosol from perilipin-2-expressing HEK293 cells provided a source of partially purified perilipin 2 [34]. While perilipin 2 efficiently targets to LDs in this cell line, a significant pool of undegraded protein remains in the cytosol [35]. Perilipin-2-expressing HEK293 cells were pelleted and resuspended in Hepes/EDTA buffer with protease inhibitors (1 μg/mL pepstatin, 1 μg/mL leupeptin, 100 μg/mL PMSF), then homogenized and lysed with a 25-g needle. Lysed cells were adjusted to 10% sucrose then loaded in a centrifuge tube and overlaid with Hepes/EDTA buffer. Samples were centrifuged at 280,000 × *g* at 4 °C for 3 h. Perilipin-2-enriched cytosol was collected from the void volume, combined with the LUVs, and incubated in an orbital mixer at room temperature for 1 h. Samples were adjusted to 25% sucrose, loaded into a centrifuge tube, overlaid with 15% sucrose in Hepes/EDTA buffer, and centrifuged at 280,000 × *g* at 4 °C for a minimum of 3 h. Floating LUVs were collected with a 22-g needle. As floating fractions were visibly turbid, we used a Nanodrop 2000 spectrophotometer to compare concentrations of collected LUVs and diluted samples when necessary to achieve overlapping spectra. Perilipin 2 was detected in floating fractions of equivalent concentrations by SDS-PAGE and immunoblotting. 

### 2.10. Cell Culture 

NIH 3T3 mouse fibroblasts, provided by Sabrice Guerrier (Millsaps College, Jackson, MS, USA) or obtained from ATCC, were cultured in DMEM (Gibco, #11965) supplemented with 10% bovine calf serum (Fisher, 41400-045, Hampton, NH, USA) and 1% penicillin/streptomycin (Gibco). AML12 cells, derived from mouse hepatocytes, were provided by Doug Mashek (University of Minnesota, Twin Cities, MS, USA) and cultured in DMEM supplemented with 10% FBS (Atlanta Biologicals, Atlanta, GA, USA), 1% penicillin/streptomycin, and 0.1% insulin-transferrin-selenium (Fisher, 41400-045, Hampton, NH, USA). For choline depletion, media was prepared with choline-deficient DMEM (a modification of Gibco #11965 formulated without choline chloride). Where indicated, media was additionally supplemented with 500 µM oleate complexed with BSA as described [36]. 

### 2.11. Isolation of LDs from Cultured Cells 

NIH 3T3 and AML12 cells were cultured with 500 µM oleate with or without choline for 48 h. The cells were washed and scraped into cold PBS, pelleted, and resuspended in Hepes/EDTA buffer with protease inhibitors. Cell suspensions were incubated on ice for 10 min and lysed by multiple passages through a 25-g needle. Cell lysates were overlaid with Hepes/EDTA buffer and centrifuged at 275,000 × *g* at 4 °C for at least 30 min. Floating LDs were transferred to a second centrifuge tube, adjusted to 25% sucrose, overlaid with Hepes/EDTA buffer and centrifuged at 275,000 × *g* at 4 °C for at least 30 min. Floating LDs were collected and spun in a microcentrifuge for 20 min at 4 °C. Removal of the infranatant increased the concentration of the LD samples. Total protein levels in each LD sample were determined with the Bradford protein assay [37]. Phosphate levels were determined using the method of Ames and Dubin [38]. 

To measure PC and PE content of isolated LDs, lipids were extracted as described by Bligh and Dyer [39]. Lipids were separated on silica plates (Analtech) with chloroform:methanol:25% ammonia (50:25:6) and visualized with iodine vapor. PC and PE bands from the lipid extract were identified by comparison to standards (Avanti Polar Lipids). The pixel intensity of each band was determined with ImageJ. 

### 2.12. SDS-PAGE and Immunoblotting of Proteins from Cultured Cells

Equivalent amounts of LUVs or total LD associated proteins were resolved on 10% SDS-PAGE gels. Proteins were transferred to nitrocellulose membrane and resolved with rabbit polyclonal antibodies to perilipin 2 (Novus Biologicals) or perilipin 3 (Nathan E. Wolins, Washington University, St. Louis, MO, USA) and peroxidase-conjugated secondary antibodies (Sigma). Bands were visualized with Supersignal West Femto ECL reagent (Fisher) and a FotoDyne FOTO/Analyst Luminary/FX workstation. 

## 3. Results

### 3.1. Effect of Ethanol on Liver Phenotype

**Figure 1:**
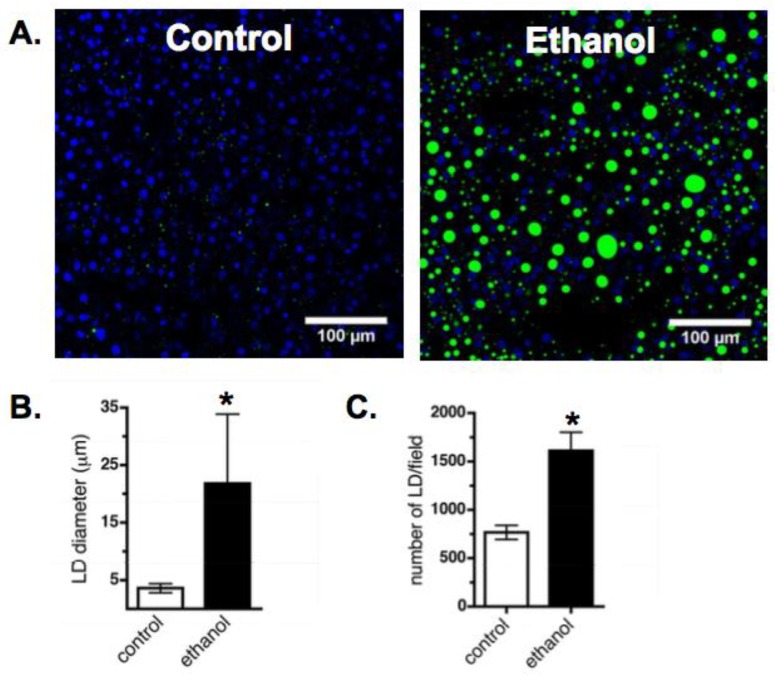
Male Wistar rats on an ethanol-enriched Lieber–DeCarli diet develop fatty liver. (**A**) Confocal images of representative liver sections stained with BODIPY (green, LDs) and DAPI (blue, nuclei). (**B**,**C**) Chronic ethanol exposure increased the diameter and number of hepatic LDs. Values are mean ± SEM from nine fields of view of liver sections from five control and five ethanol-fed animals. Data were analyzed by ANOVA followed by Tukey’s test for specific comparisons between means (*, *p* < 0.05).

**Figure 2:**
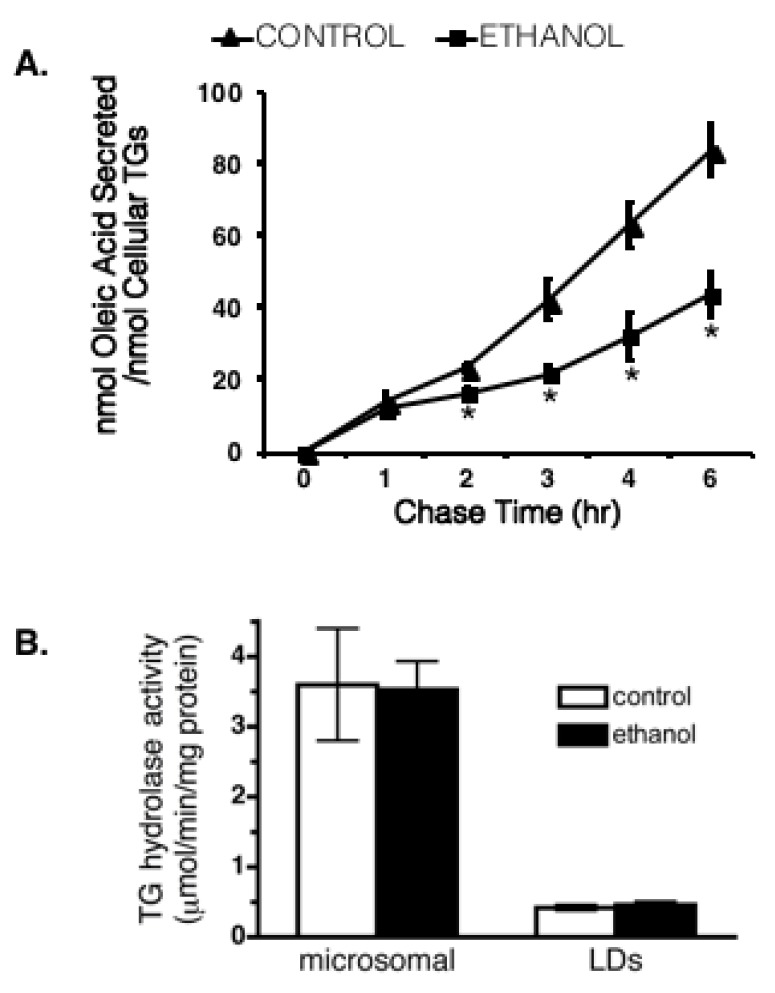
Chronic ethanol exposure decreased lipolysis from isolated hepatocytes. (**A**) The efflux of radiolabeled oleic acid from hepatocytes isolated from control or ethanol-fed rats. Values are mean ± SEM (*, *p* < 0.05; *n* = 5). (**B**) Triacylglycerol hydrolase (TGH) activity in microsomal and lipid droplet (LD) fractions.

Five to seven weeks on an ethanol-enriched Lieber–DeCarli diet led to dramatic increases in hepatic lipid accumulation in male Wistar rats (Figure 1A). Measurements of BODIPY-stained LDs in frozen liver sections from control and ethanol-fed rats demonstrated an increase in both LD size (Figure 1B) and number (Figure 1C). We also detected differences in hepatic lipid metabolism following ethanol feeding. Hepatocytes isolated from ethanol-fed rats displayed a decrease in the rate of lipolysis (Figure 2A). Since up to 70% of the triglycerides packaged and secreted by hepatocytes in very-low-density lipoproteins (VLDLs) are derived via lipolysis of preformed triglyceride stores in LDs [40,41,42], the approximate 50% reduction in lipolysis observed in this study is consistent with our previous report showing a decreased in vivo VLDL secretion rate in the ethanol-fed rats compared to control rats [43]. 

Triacylglycerol hydrolase (TGH) is a major lipase in hepatocytes that has been shown to mobilize LD triglycerides for VLDL assembly [44,45,46]. Thus, we hypothesized that the ethanol-induced reduction in lipolysis may be a consequence of reduced hepatic TGH activity in these rats. However, we observed no difference in hepatic TGH activity between control and ethanol-fed animals (Figure 2B). 

### 3.2. Composition of Isolated Hepatic Liver Droplets

**Figure 3:**
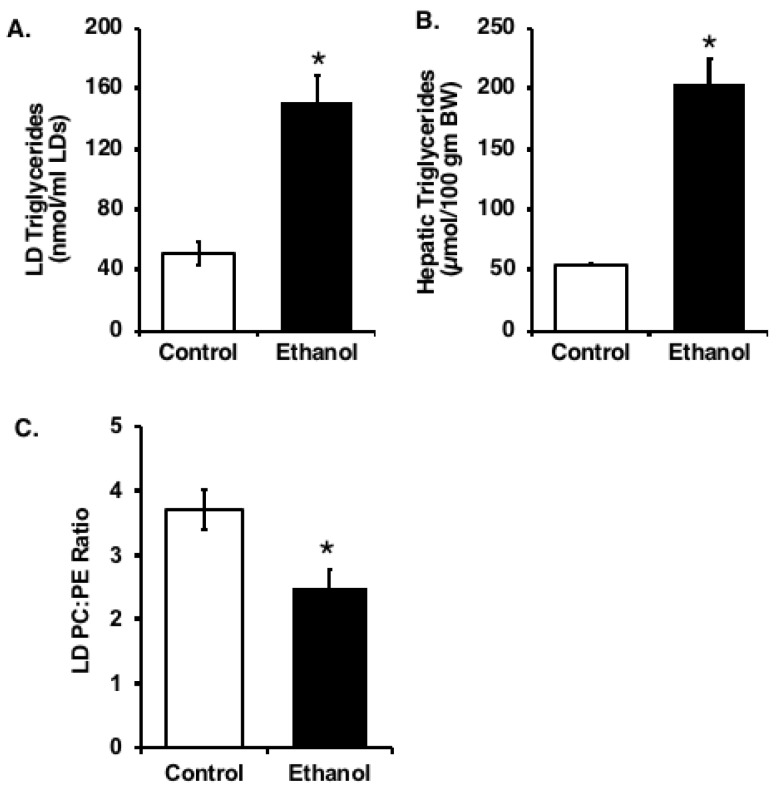
Chronic ethanol exposure increases the triglyceride content in the liver and alters the lipid composition of hepatic LDs. (**A**) Triglyceride levels in isolated LD fractions. (**B**) Total liver triglyceride levels expressed relative to body weight (BW). (**C**) Relative phospholipid levels in isolated LD fractions. Values are mean ± SEM (*, *p* < 0.05; *n* = 5).

To better understand the link between ethanol consumption and lipid accumulation, we examined the protein and lipid composition of hepatic LDs from control and ethanol-fed rats. We detected an increase in the amount of triglyceride in the hepatic LDs isolated from ethanol-fed rats (Figure 3A). This was consistent with the greater overall accumulation of triglycerides in the livers of these animals (Figure 3B). Additionally, the amount of PC relative to PE was significantly decreased in the LD fractions from ethanol-fed rats compared to hepatic LDs from control rats (Figure 3C). This difference is likely because of the reduction in the generation of PC via PEMT-catalyzed methylation of PE due to the lowering of the hepatocellular SAM:SAH ratio in the ethanol-fed (2.49 ± 0.22) compared to control (4.40 ± 0.59) rats as reported previously [23,27].

**Figure 4:**
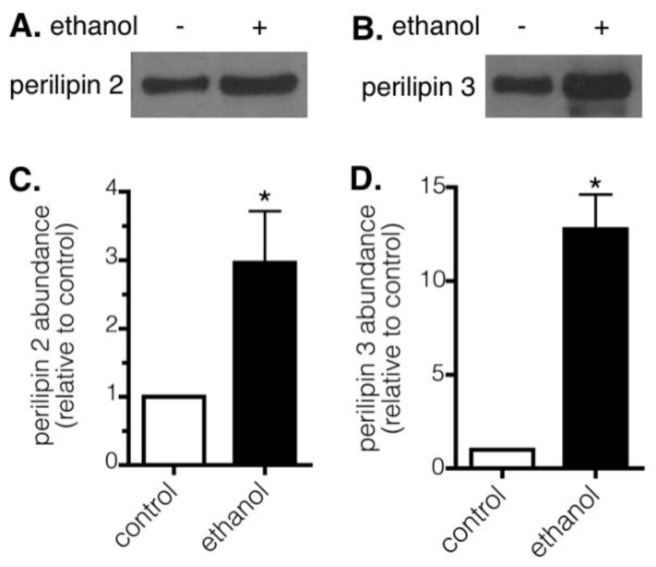
Chronic ethanol exposure alters the protein composition of hepatic LDs. Levels of perilipin 2 (**A**,**C**) and perilipin 3 (**B**,**D**) in equivalent amounts (per gram) of liver tissue were determined by SDS-PAGE and Western blotting. (**A**,**B**) Representative Western blots from control (−) or ethanol (+) treated rats are shown. (**C**,**D**) Summary data are average values relative to the control ± SEM from five experiments. Asterisks indicate values for ethanol-treated rats are statistically different than 1.0 (*, *p* < 0.04, Wilcoxon signed-rank test).

SDS-PAGE and Western blotting were used to determine levels of prominent lipid droplet proteins in LD fractions isolated from the livers of control and ethanol-fed rats. Similar to previous reports [18], LD fractions from the livers of ethanol-fed rats contained elevated levels of perilipin 2 and 3 (Figure 4).

### 3.3. In Vitro Analysis of Perilipin 2 Binding

Many organelles use special lipids (e.g., phosphoinositides, phosphatidylserine, phosphatidic acid) for mediating protein targeting to membranes [47,48]. Whether LDs utilize such a strategy for recruiting surface proteins is unknown. Since the phospholipid ratio (PC and PE) and surface protein association (perilipins 2 and 3) in hepatic LDs are both altered by ethanol exposure, we considered the possibility that these major LD surface phospholipids played a role to recruit LD proteins. To test this conjecture, we utilized an in vitro binding assay previously developed to explore perilipin 2 targeting to lipid droplet surfaces [34]. 

The in vitro binding assay requires a large amount of soluble protein. This is difficult as LD proteins, including the perilipin family members, frequently precipitate upon purification [49]. To circumvent this problem, we used the cytosol from a perilipin 2 overexpresssing cell line as a source of soluble protein [35]. We have previously shown that perilipin 2 from this cytosolic fraction can bind to large LUVs and that this interaction depends on the acyl chain composition of the surface phospholipids. Here, we extended that work to determine if the headgroups of the surface phospholipids also influenced perilipin 2 binding. 

**Figure 5:**
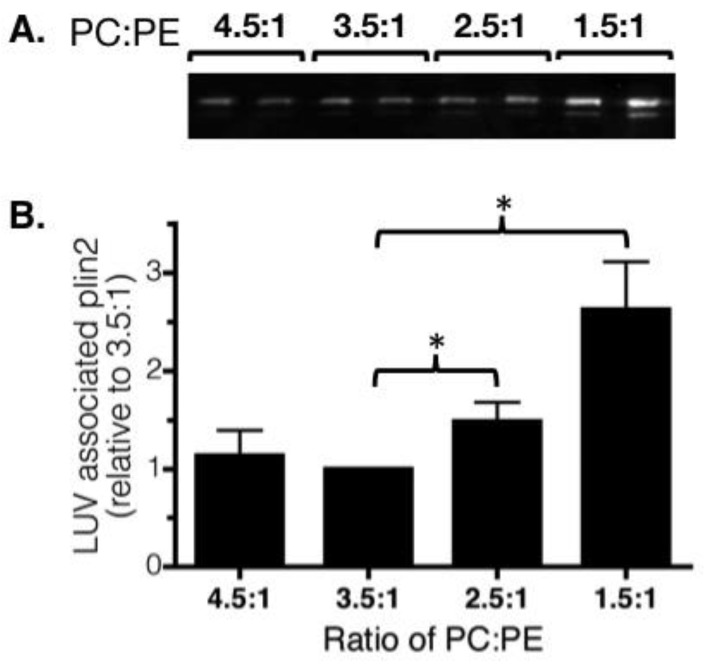
A decrease in the amount of phosphatidylcholine (PC) relative to phosphatidylethanolamine (PE) promoted perilipin 2 binding to large unilamellar vesicles (LUVs). (**A**): A representative Western blot of perilipin 2 in equivalent amounts of floating LUV fractions is shown. (**B**): Perilipin 2 abundance in LUV fractions was quantified. Data are expressed relative to the PC:PE ratio detected in hepatic LDs from control animals (3.5:1) and are mean values ± SEM from six to seven experiments. (*, *p* < 0.02, Wilcoxon signed-rank test).

We constructed LUVs that mimicked the PC:PE ratios observed in the livers of control and ethanol-fed rats and incubated the LUVs with perilipin 2 from the cytosolic fraction of perilipin 2 overexpressing HEK 293 cells [35] as previously described [34]. Next, unbound perilipin 2 was separated from the LUVs by sucrose gradient centrifugation. We compared the amount of perilipin 2 that fractionated with the LUVs of each phospholipid composition (Figure 5). These experiments showed that the relative levels of PC and PE influenced the amount of perilipin 2 bound to LUVs. Specifically, perilipin 2 association increased as the ratio of PC to PE decreased. This difference was significant for PC:PE ratios that modeled the hepatic lipid droplets of control (3.5:1) and ethanol-fed (2.5:1) rats. 

### 3.4. Manipulation of LD Phospholipid Levels in Cultured Cells

Our in vitro experiments support the hypothesis that the relative levels of PC and PE influence perilipin 2 binding. We sought to further explore the relationship between LD phospholipid ratios and associated protein levels in a cell culture system where we could manipulate LD composition while maintaining the complexity of in vivo LD form and function. We chose to manipulate phospholipid levels directly rather than induce changes through ethanol supplementation. With this approach, we aimed to confirm that decreasing PC content relative to PE in the LD surface monolayer would be able to influence LD protein composition on its own, independent of other factors generated by ethanol consumption. 

**Figure 6:**
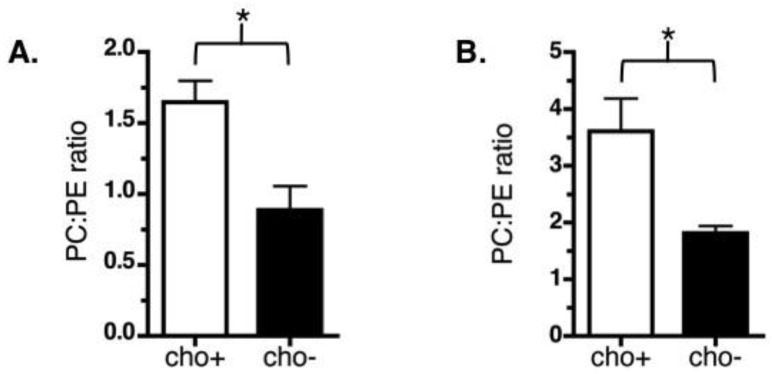
Choline deficiency significantly reduces the PC:PE ratio of LD phospholipid monolayers. NIH 3T3 (**A**) or AML12 (**B**) cells were grown with 500 μM oleate in control (cho+) or choline-deficient (cho−) media for 48 h. LDs were isolated and phospholipids were extracted, then separated and identified by thin layer chromatography. Relative PC:PE levels are shown. Data are the average and SEM of four experiments (*, *p* < 0.03, unpaired *t*-test, two-tailed).

We altered the relative amount of PC and PE on LDs in cultured NIH 3T3 fibroblasts by diminishing the amount of available choline. In most tissues, over 70% of PC is synthesized directly from choline via the Kennedy pathway and up to 95% of available choline is used in this synthesis of PC [50]. We therefore hypothesized that culturing cells in choline-deficient media for 48 h would decrease intracellular PC through elimination of the primary precursor. Moreover, we hypothesized that the decrease in intracellular PC would alter phospholipid surfaces throughout the cell, including the LD surface. To test this strategy, LDs were isolated from control and choline-deficient NIH 3T3 cells and the phospholipid components were separated by thin layer chromatography and quantified. LDs from choline-deficient NIH 3T3 cells had less LD-associated PC relative to PE when compared with LDs from control cells (Figure 6A). We used a similar approach to attempt to alter phospholipid levels in AML12 cells, a mouse hepatocyte cell line. AML12 cells also showed decreased PC relative to PE on LDs following 48 h in choline-deficient media (Figure 6B). Thus, choline depletion is an effective general strategy for altering the phospholipid composition of LDs in cultured cells. 

### 3.5. Manipulation of LD Phospholipid Levels in Cultured Cells Alters Protein Binding

**Figure 7:**
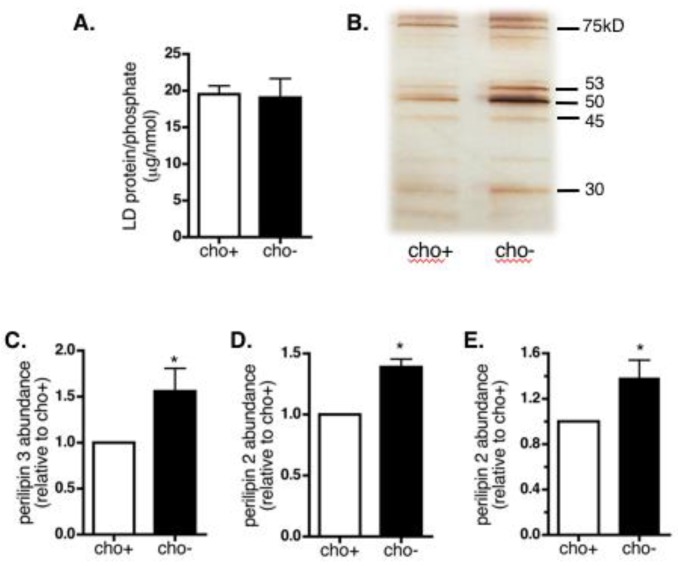
LD protein composition in choline-deficient cells. LDs were isolated from NIH 3T3 (**A**–**D**) or AML12 (**E**) cells grown in oleate-supplemented media with (cho+) or without (cho−) choline. (**A**) Total levels of protein in LD fractions are shown relative to levels of phosphate in those same fractions. Data are averages ± SEM from five independent experiments. (**B**) Equivalent amounts of total proteins from LD fractions were separated by SDS-PAGE and visualized by silver stain. A representative gel is shown. (**C**–**E**) Levels of perilipin 2 and perilipin 3 in equivalent micrograms of total LD proteins were determined by SDS-PAGE and Western blotting. Data are average values relative to the control ± SEM from five to seven experiments. Asterisks indicate cho- is statistically different than 1.0 (*, *p* < 0.04, Wilcoxon signed-rank test).

We next aimed to determine whether decreasing the amount of PC relative to PE on LDs in cultured cells would change the number or identities of associating proteins. We isolated LDs from NIH 3T3 cells after 48 h in oleate-supplemented control or choline-deficient media. When we examined LD protein/LD phosphate content, we observed no significant difference in the total amount of LD protein in control versus choline-deficient cells (Figure 7A). However, analysis of LD proteins by SDS-PAGE and silver staining indicated that choline deficiency altered the LD association of a subset of LD proteins (Figure 7B). 

We used SDS-PAGE and Western blotting of LD proteins from control and choline-deficient cells to look specifically at LD association of perilipin 2 and 3, proteins that were abundant in hepatic LDs from the ethanol-fed rats. Our results indicated a 1.5-fold increase in perilipin 3 association with LDs in choline-deficient NIH 3T3 cells (Figure 7C). We also detected a 1.4-fold increase in association of perilipin 2 with LDs from choline-depleted NIH 3T3 cells compared to LDs of control cells (Figure 7D). Similar experiments in choline-deficient AML12 cells also demonstrated an increase in LD-associated perilipin 2 (Figure 7E). These results provide support to the hypothesis that decreasing the amount of LD PC relative to PE alters LD association of specific proteins. 

## 4. Discussion

Analysis of LDs in a rat model of AFLD provides new information about the progression of this disease. Rats on an ethanol-enriched diet for 5–7 weeks had a massive overaccumulation of hepatic LDs with lower levels of lipolysis. The decrease in lipolysis likely negatively impacted VLDL secretion, as was previously reported [43]. The hepatic LDs from ethanol-fed rats showed differences in phospholipid and protein composition when compared with LDs isolated from control animals. Specifically, hepatic LDs from ethanol-fed rats showed a decrease in the PC:PE ratio of surface phospholipids compared to controls. We believe that the changes to phospholipid levels were caused by our previously reported impairment in PEMT activity [23,27]. Inhibition of PEMT likely occurs in response to ethanol-induced elevation of hepatocellular SAH and a reduction in the SAM:SAH ratio.

Our data support a model where the relative amounts of PC and PE on the surface of LDs impact the association of specific LD proteins. We detected more perilipin 2 and 3 in LD fractions from ethanol-fed animals where phospholipid levels were also changed. However, because hepatocytes from ethanol-fed rats also had an elevated number of LDs, we cannot definitively attribute the higher levels of perilipin 2 and 3 to the altered phospholipid content of LDs. Instead, we sought an alternate approach to determine the effect of changes in LD phospholipid levels. Notably, while the total level of LD protein was not changed, we measured an increase in perilipin 2 and 3 on LDs in NIH 3T3 and AML12 cells cultured under conditions that altered LD phospholipid levels. Moreover, we demonstrated a direct link between PC:PE composition and perilipin 2 binding in an in vitro assay that detected protein association with phospholipid vesicles. Both of these results suggest that changes to LD surface properties impact protein binding. 

Evidence is emerging that the unique biophysical properties of the LD surface facilitate localization of LD proteins. Some LD proteins likely access the LD from the endoplasmic reticulum; others are translated in the cytosol and associate with LDs via amphipathic alpha helices or other hydrophobic domains [9]. Proteins that localize to LDs through amphipathic alpha helices may rely on “packing defects” at the LD surface to assist in association and folding [51]. Packing defects describe areas of the LD surface where the underlying hydrocarbons of the phospholipid acyl chains or core neutral lipids are exposed to the surrounding aqueous environment. Two recent reports describe the prevalence of packing defects at the LD surface [51,52]. Our previous experiments suggest that perilipin 2 localizes to LDs through a mechanism involving LD surface packing defects [34]. We demonstrated that perilipin 2 association with lipid vesicles was disrupted by increasing the proportion of surface phospholipids with saturated acyl chains, a change that also increased surface lipid packing and likely decreased access to the underlying hydrophobic lipids.

In this paper, we identify changes to the PC:PE composition of the LD surface. We hypothesize that this change in LD surface composition also increases LD surface packing defects. PC is a cylindrical phospholipid and as such may form a surface that shields underlying hydrophobic lipid from interacting with surface proteins. On the other hand, the smaller headgroup of PE may allow additional interactions between surface proteins and hydrophobic lipids. Proteins like perilipin 2 and 3, that interact with LDs through amphipathic helical domains [53], may associate more readily with a surface enriched for PE rather than PC as there would be more opportunity for interaction between the hydrophobic amino acid side chains and underlying hydrophobic lipid. This work is therefore consistent with the model shared by Prevost and Sharp et al., whereby packing defects in the phospholipid monolayer at the LD surface assist in the association and folding of amphipathic alpha helical domains of LD proteins [51]. 

There is ample evidence linking perilipin family proteins to LD growth [12]. Perilipin 2 association with LDs has been shown to promote LD accumulation [54,55,56,57,58] by reducing triglyceride turnover [35], and perilipin 2 knockout mice are protected from hepatic steatosis [19,20]. Thus, changes to the amount of perilipin 2 on LDs may directly contribute to the growth and accumulation of hepatic LDs. Ongoing experiments in our laboratory aim to identify additional proteins that preferentially bind LDs enriched for PC and could contribute to LD accumulation. Perilipin 5 is a candidate protein given its sequence similarity to perilipin 2. Perilipin 5 expression is elevated with hepatic steatosis and its ablation decreases lipid accumulation in the liver [59].

Changes in phospholipid composition may impact LD size and number through pathways that do not involve altering the LD proteome. For example, LDs that lack sufficient PC to shield underlying hydrophobic lipids fuse, thereby creating super-sized LDs [60,61]. Moreover, blocking PC synthesis promotes lipid droplet accumulation through SREBP-1-dependent gene transcription [62]. The relative contribution of each of these pathways is unknown but the end result is clear; a change to the relative amount of phosphatidylcholine on LDs appears to promote LD growth. 

Our rat model of AFLD shows some similarities to other models of pathophysiological changes to hepatic lipid metabolism. A choline-deficient diet is widely used to promote liver steatosis in animal models, whereas choline supplementation reverses the liver steatosis observed in rats on a high-fat diet [63]. A decrease in hepatic PC relative to PE also correlates with the development of hepatic steatosis in a mouse model of nonalcoholic fatty liver disease [64]. Collectively, these studies further support a link between changes to LD phospholipid levels and overaccumulation of LDs in animal models.

The link between LD surface content and LD growth and accumulation is further supported by several studies in cultured cells. Knockdown of enzymes involved in PC biosynthesis leads to the formation of large LDs in *Drosophila* S2 cells and murine bone-marrow-derived macrophages [60,61]. Horl et al. demonstrated a significant decrease in the ratio of PC to PE on LDs during differentiation of 3T3-L1 adipocytes [65]. Moreover, inhibition of PC synthesis in this cell line by suppression of PEMT expression increased lipid accumulation, decreased basal lipolysis, and led to a decrease in LD association of perilipin A, the predominant perilipin protein in adipocytes. This work suggests that the correlation between LD phospholipid content and LD accumulation is not specific to the cell types included in this study but plays a more basic role in lipid accumulation in multiple tissues. 

As a dynamic organelle, the composition of an LD responds to changing nutrient and hormone levels [66,67]. Our work suggests that specific changes to the phospholipid composition of hepatic LDs can impact its function by modifying the association of specific LD proteins, which in turn affects the breakdown of the LD triglyceride stores, the rate of formation and secretion of VLDL, and, ultimately, lipid accumulation. Therefore, careful examination of features of the LD surface and, more specifically, the way in which LD composition changes under pathophysiological conditions may contribute to our understanding of multiple diseases that involve overaccumulation of lipid droplets including AFLD and nonalcoholic fatty liver disease.
